# Influence of Graphene Platelet Aspect Ratio on the Mechanical Properties of HDPE Nanocomposites: Microscopic Observation and Micromechanical Modeling

**DOI:** 10.3390/polym12081719

**Published:** 2020-07-31

**Authors:** Evangelia Tarani, Iouliana Chrysafi, Alfréd Kállay-Menyhárd, Eleni Pavlidou, Thomas Kehagias, Dimitrios N. Bikiaris, George Vourlias, Konstantinos Chrissafis

**Affiliations:** 1 Physics Department, Aristotle University of Thessaloniki, GR541 24 Thessaloniki, Greece; etarani@physics.auth.gr (E.T.); iochrysa@physics.auth.gr (I.C.); elpavlid@auth.gr (E.P.); kehagias@auth.gr (T.K.); gvourlia@auth.gr (G.V.); 2 Institute of Materials Science and Environmental Chemistry, Research Centre for Natural Sciences, Hungarian Academy of Sciences, Magyar Magyar Tudósok Körútja 2, 1117 Budapest, Hungary; amenyhard@mail.bme.hu; 3 Laboratory of Polymer Chemistry and Technology, Department of Chemistry, Aristotle University of Thessaloniki, GR541 24 Thessaloniki, Greece; dbic@chem.auth.gr

**Keywords:** GNPs, HDPE, nanocomposites, mechanical properties, Takayanagi model, Ji model, fractography

## Abstract

A series of high-density polyethylene nanocomposites filled with different diameter sizes (5, 15, and 25 μm) of graphene nanoplatelets at various amounts (0.5–5 wt.%) are prepared by the melt-mixing method. The effect of diameter size and filler content on the mechanical properties is reported, and the results are discussed in terms of morphology and the state of dispersion within the polymer matrix. The measured stiffness and strength of the nanocomposites were found to be mainly influenced by the filler aspect ratio and the filler-matrix adhesion. Fractography was utilized to study the embrittleness of the nanocomposites, and the observations revealed that a ductile to brittle transition is caused by a micro-deformation mechanism change in the nanocomposites. Several micromechanical models for the prediction of mechanical properties of nanocomposites, taking into consideration filler aspect ratio, percolation effect, and interphase regions, are considered. The three-phase model proposed by Ji accurately predicts the stiffness of graphene nanoplatelets with a higher diameter size, while Takayanagi modified model II was found to show good agreement with the experimental results of smaller ones at low filler content. This study demonstrates that the diameter size of the filler plays a central role in determining the mechanical properties.

## 1. Introduction

There has been a growing interest in the nanocomposites applied in thermally conductive polymeric materials. Conducting polymers that are highly conductive and electrochemically active have been focused on some promising applications such as solar cells, sensors, energy storage devices, and heat pump pipes [1]. Polyethylene (PE) is a low-cost semi-crystalline polymer that shows good thermal and mechanical properties and high chemical resistance. Therefore, it can be used effectively in various applications in automotive, films, pipes, bottles, tubes, and cable jacketing [2]. PE exhibits an orthorhombic structure Pnam space group, with two polymeric chains per unit cell. The lattice parameters of PE are reported to be a = 7.43 Å, b = 4.94 Å, and c = 2.55 Å [3]. In certain areas, PE use is limited by its own low mechanical and gas barrier properties, poor heat resistance, and low thermal and electrical conductivities, among others [4]. To overcome these shortcomings, various fillers such as carbon black, carbon nanotubes, graphene, graphene oxide, graphene nanoplatelets, and others, have been introduced.

Graphene nanoplatelets (GNPs) are platelet-like graphite nanocrystals with a platelet thickness in the range of 0.35–100 nm containing multiple graphene layers [5]. It is known that among different carbon fillers, the one with a platelet shape offers advantages over other morphologies: (i) they can be dispersed uniformly, compared to CNTs; (ii) they possess a high surface area improving load-transfer properties; (iii) GNPs, with a two-dimensional lattice of sp^2^- bonded carbon, may enable heat transferred in a plane, which can further increase the thermal performance of polymers; and (iv) additional advantages of GNPs over other types of fillers are its moderate cost and ease of processing [6,7,8,9].

Many studies have reported the benefits of adding graphene and its derivatives in the PE matrix to reinforce the thermal, electrical, and mechanical properties of the PE matrix. However, several reports have been reported on the preparation method of PE/graphene nanocomposites [10,11,12,13,14], the improvement in the interfacial interaction between PE-functionalized GNPs [15,16,17,18,19], the dispersion and alignment of graphene in the composite samples [20,21], and the construction of the hybrid network structures of graphene with other conductive fillers [10,22,23]. Although the GNPs size is an important factor, only a few works have studied the GNP’s size effect on the mechanical properties of High-Density Polyethylene (HDPE) nanocomposites [24,25,26].

Conventional micromechanical models are used to predict the effective elastic modulus of HDPE/graphene composites [27,28]. In these models, the effective properties are obtained based on the matrix properties, the fillers volume/weight fraction, and the aspect ratio. However, some other parameters should be included such as the interaction between the filler and matrix and the percolation effect. On the one hand, the large surface area of the nanofillers increases the mobility of the surrounding polymer chains to many gyration radii, forming the interphase regions [26]. On the other hand, the percolation threshold has been reported to affect the mechanical properties of polymer nanocomposites containing conductive fillers [29,30]. The high surface area and aspect ratio of GNPs leads to much lower values for the percolation threshold of GNP nanocomposites compared to conventional fillers such as carbon fibers and carbon black. Some authors have reported that the percolated structure may be formed at a volume fraction below the theoretical threshold, due to the connectivity among the interphase regions [31,32]. These findings can provide detail on the optimal shape and geometry of conducting fillers to maximize the benefits of conducting fillers and minimizing the percolation threshold.

In our previous works on HDPE/GNP nanocomposites [33,34,35], it was found that GNP M25 with the larger diameter size reduced the chain mobility in the semi-crystal HDPE matrix causing the degradation of the HDPE to be retarded. The large contact areas and continuous paths increased the thermal conductivity values. HDPE/M25 nanocomposites showed slower crystallization and increased rheological properties because of the formation of larger aggregates compared to HDPE nanocomposites filled with a small diameter size. Therefore, many factors are responsible for the thermal performance improvement of nanocomposites, such as filler size, geometry, dispersion, and interaction with the matrix. All these parameters must be considered to better understand the effect of the filler size on the mechanical properties of the HDPE matrix.

For the continuing effort to understand the mechanical properties of HDPE/GNP nanocomposites, we have undertaken a comprehensive study of microscopic observation and micromechanical modeling. For this reason, HDPE/GNP nanocomposites have been produced by the melt-mixing method using three different diameter sizes, 5, 15, and 25 μm with a filler content of 0.5–5 wt.%. To better understand the mechanical properties, a morphological study of GNPs into the HDPE matrix was performed by transmission electron microscopy and scanning electron microscopy. Additionally, dynamic mechanical analysis of the HDPE/GNPs nanocomposites was performed to determine the thermo-mechanical properties, in other words, storage and loss moduli. The mechanical properties of HDPE/GNP nanocomposites were characterized by tensile tests. Various micromechanical models were tested and compared with experimental results considering the dispersed and agglomerated GNPs in the HDPE matrix and the effect of the percolation threshold on the elastic modulus of the composites. Finally, scanning electron microscopy was used to analyze the fracture surfaces of the HDPE/GNP nanocomposites after tensile measurements.

## 2. Materials and Methods

HDPE under the trade name Luminece mPE M5510 EP was supplied by Total Petrochemicals (Feluy, Belgium). It has a melt flow index of 0.28 g/10 min and 0.955 g cm^−3^ density. GNPs with an average thickness of 8 nm were supplied by XG Sciences Inc., Lansing, Mich., USA, including three different average diameters: GNPs with an average platelet diameter of 5 μm (GNPs M5), 15 μm (GNPs M15), and 25 μm (GNPs M25). Additionally, the average surface area ranges from 120 to 150 m^2^/g. The bulk density of all GNPs is reported to be 2.2 g/cm^3^.

HDPE/GNP nanocomposites containing various loading levels ranging from 0.1 to 5 wt.% (0.5, 1, 2.5, 3, and 5 wt.% of GNPs) were prepared by melt mixing in a reomixer ((model 600), Haake-Buchler Instruments Itd., Saddle Brooke, NJ, USA) equipped with roller blades and a mixing head with a volumetric capacity of 69 cm^3^. For this set of samples, mixing was performed at 200 °C, for 600 s, using a torque speed of 35 rpm. This was followed by the prepared materials being hot pressed using an Otto Weber, Type PW 30 hydraulic press (Paul-Otto Weber GmbH, Remshalden, Germany) connected with an Omron E5AX temperature controller (Omron, Kyoto, Japan), at a temperature of 180 ± 5 °C to prepare films of different thicknesses, appropriate for each type of the following measurements. Figure S1 of the Supplementary Information shows a schematic diagram of the melt-mixing process of HDPE/GNPs nanocomposites. The nanocomposite samples are referred to here as HDPE/xM5, HDPE/xM15, and HDPE/xM25, where x is GNP content in wt.%.

The morphological and structural properties of GNPs were investigated by transmission electron microscopy (TEM) and selected area electron diffraction (SAED) experiments using a Jeol JEM 1010 electron microscope, Jeol Ltd., Akishima, Japan, operated at 100 kV. TEM specimens were prepared by sectioning the samples in a Leica UCT Ultracut (Leica Microsystems, Buffalo Grove, IL, USA) ultramicrotome and collecting thin sections on 400-mesh Cu grids.

Scanning electron microscopy (SEM) micrographs of the failure surfaces of HDPE/GNP nanocomposites after tensile testing were studied by using a SEM JEOL JSM-6390LV model (Jeol Ltd., Akishima, Japan). The specimens were carbon coated to provide good conductivity of the electron beam and the operating conditions were accelerating voltage 20 kV, probe current 45 nA, and counting time 60 s. To prepare the sample, a thin strip (3 mm wide, 3.3 mm thick, and 5 mm tall) was cut from the specimen so that the fracture surface could be viewed.

Dynamic mechanical analysis (DMA) experiments were performed by a Pyris Diamond DMA, (Perkin Elmer, Waltham, MA, USA). The measurements were carried out in tensile mode at 2 Hz frequency and 10 μm deformation amplitude. The temperature range varied from −150 to 130 °C, at a heating rate of 2 °C/min in an atmosphere of N_2_ (>99.9%). The temperature dependent behavior was studied by monitoring changes in force and phase angle, keeping the amplitude of oscillation constant. Samples of approximately 50 mm length, 5 mm width, and 3 mm thickness were used for the DMA study. The specimens’ dimensions were measured accurately using a Profi micrometer and three specimens were manufactured for each case. The study of the mechanical parameters, like storage modulus (E’), loss modulus (E’’) and loss factor (tanδ), along with the Tg temperature (glass transition) were evaluated. Figure S2 of the Supplementary Information shows the principle of DMA measurements for HDPE/GNP nanocomposites.

Measurements of the tensile mechanical properties of the prepared nanocomposites were performed on an Instron 3344 dynamometer, in accordance with ASTM D638 (Norwood, MA, USA), using a crosshead speed of 50 mm/min. Dumb-bell-shaped tensile test specimens (central portions 5 mm × 0.5 mm thick, 22 mm gauge length) were cut in a Wallace cutting press. Figure S3 of the supplementary information shows the tensile tests experiment setup for HDPE/GNPs nanocomposites. The values of tensile strength at the yield point and break, elongation at break, and the Young modulus were calculated from stress–strain curves. Five specimens for each type of HDPE/GNP nanocomposite were tested and the results were averaged to obtain a mean value to ensure reproducibility.

## 3. Results and Discussion

### 3.1. Theoretical Background

**Figure 1:**
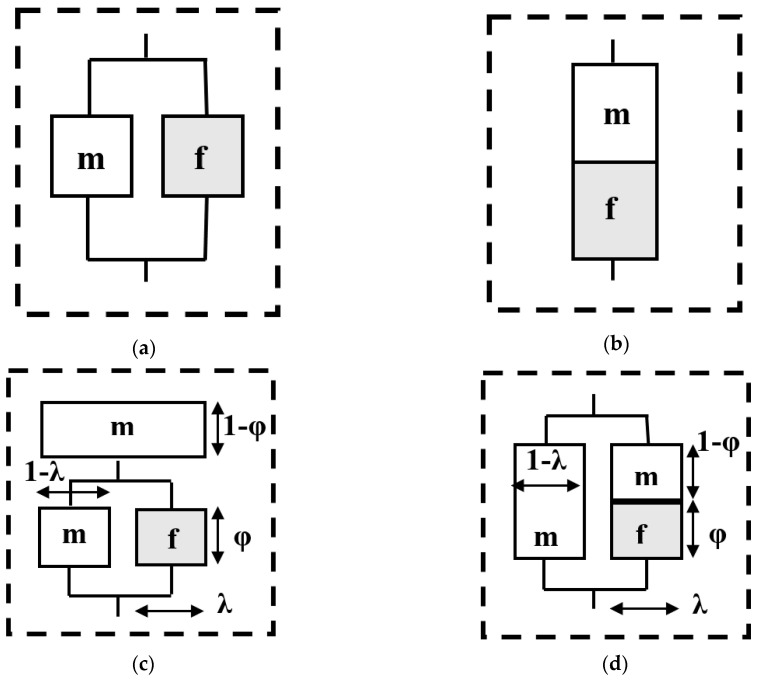
Models for a two-phased system; (**a**) an iso-strain model, (**b**) an iso-stress model, (**c**) Takayanagi I (series–parallel), and (**d**) Takayanagi II (parallel–series) [36].

In this study, micromechanical-based models have been used to analyze the effect of filler size on the mechanical properties of the HDPE matrix and compare them with the experimental data through tensile tests. The popular micromechanical models used for the prediction of modulus of elasticity are Voigt upper bound, Reuss low bound model, Halpin–Tsai model, Takayanagi model, and Ji model [36,37,38,39,40,41]. To determine the Young modulus, the study of two distinct situations where the particles and matrix are subjected to either uniform strain or uniform stress is necessary. There are parallel and series models for polymers with two separate phases corresponding to the upper and lower bounds of the tensile property predictions, respectively. In the parallel model, (Figure 1a) an iso-strain condition (i.e., when filler and matrix have the same uniform strain under certain stress applied) exists and the total stress on the system is the sum of all the stresses.The mechanical properties of the system are given by the Voigt rule of mixtures (ROM):

(1) $$E_{c}=V_{f}E_{f}+V_{m}E_{m}$$

where E_c_, E_f,_ and E_m_ are the Young modulus of the composite, filler, and matrix, respectively. V_f_ and V_m_ are the volume fraction of filler and matrix (where V_f_ + V_m_ = 1).

Figure 1b shows the case of uniform stress where the Young modulus of the composite is given by the Reuss inverse rule of mixtures (IROM) model:

(2) $$\frac{1}{E_{c}}=\frac{V_{f}}{E_{f}}+\frac{V_{m}}{E_{m}}$$

When E_f_ » E_m_, the above-mentioned equations either over-estimate or under-estimate the mechanical properties of the composites, and they are normally taken as upper and lower bounds of the Young modulus of the composite.

The general form of the longitudinal modulus developed by the Halpin–Tsai equation [42] is given by:

(3) $$\frac{E_{c}}{E_{m}}=\frac{1+\xi \cdot \eta \cdot V_{f}}{1-\eta \cdot V_{f}}$$

where the parameter η is given by:

(4) $$\eta =\frac{\frac{{Ε}_{f}}{{Ε}_{m}}-1}{\frac{{Ε}_{f}}{{Ε}_{m}}+\xi }$$

In Equation (4), the parameter ξ is a measure of reinforcement geometry depending on the filler loading and aspect ratio. The limiting values of ξ are ξ = 0 and ξ = ∞ for the prediction of the longitudinal modulus of reinforcement by a platelet filler of length *l* and thickness t. The value of ξ can be taken as 2*l*/t. Equation (3) tends to the lower, uniform stress, bound (Equation (2)) as ξ > 0 and to the upper, uniform strain, bound (Equation (1)) as the aspect ratio of the reinforcement increases and ξ > ∞ [43].

Takayanagi et al. [37,38,39] proposed a model for the tensile modulus of polymer composites based on the series and parallel models. The two-phase mechanical model consists of a homogeneous rigid discontinuous phase and a homogenous continuous matrix phase. The equation for the series–parallel Takayanagi model (Figure 1c) is given as:

(5) $${Ε}_{c}={\left[\frac{\varphi }{\lambda \cdot E_{f}+\left(1-\lambda \right)E_{m}}+\frac{1-\varphi }{E_{m}}\right]}^{-1}$$

where λ and φ are geometry factors representing phase morphology in the Takayanagi model.

Takayanagi’s parallel–series model (Figure 1d) is more suitable when the stress transfer across planes is weak. In this model, λ is the fraction of the system in series and 1 – λ is the fraction of the system in parallel. The composite modulus is:

(6) $${Ε}_{c}=\lambda \cdot {\left(\frac{\varphi }{E_{f}}+\frac{1-\varphi }{E_{m}}\right)}^{-1}+\left(1-\lambda \right)\cdot E_{m}$$

An assumption at small strain for the volume fraction is:

(7) $$V_{f}=\lambda \cdot \varphi$$

Introducing Equation (7) into Equation (6), the following expression for the composite modulus is obtained as a function of φ:

(8) $$E_{c}=E_{m}\cdot \left(1-\frac{V_{f}}{\varphi }\right)+\left(\frac{V_{f}\cdot E_{m}\cdot E_{f}}{E_{m}\cdot \varphi^{2}+\left(1-\varphi \right)\cdot \varphi \cdot E_{f}}\right)$$

**Figure 2:**
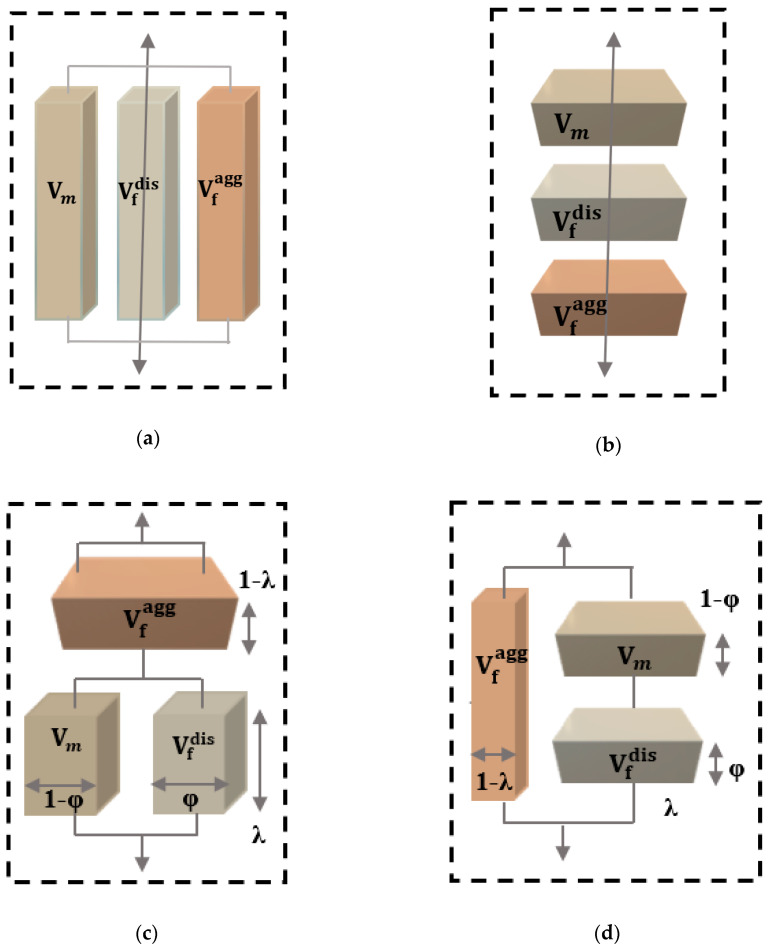
Three-phase composites in (**a**) series, (**b**) parallel, (**c**) Takayanagi model I, and (**d**) Takayanagi model II with a percolation concept [49].

Quali et al. [44] proposed a model based on the IROM by the percolation concept as:

(9) $$E=\frac{\left(1-2\cdot \Psi +\Psi \cdot V_{f}\right)E_{m}E_{f}+\left(1-V_{f}\right)\cdot \Psi \cdot E_{f}^{2}}{\left(1-V_{f}\right)\cdot E_{f}+\left(V_{f}-\Psi \right)\cdot E_{m}}$$

(10) $$\Psi =\{\begin{array}{c} 0,\;V_{f}<V_{c}\\ V_{f}\cdot {\left(\frac{V_{f}-V_{c}}{1-V_{c}}\right)}^{b},\;V_{f}\geq V_{c} \end{array}$$

where V_c_ is the least volume fraction of the filler which forms a continuous network in the nanocomposite, known as percolation threshold. The b parameter has a value of b ~0.4 in a 3-D structure. Additionally, Ψ = 0 below the percolation threshold reduces the Quali model to the IROM. Although this model was extensively applied to polymer nanocomposites, it cannot predict the mechanical behavior of polymer nanocomposites above the percolation threshold [45]. Therefore, the composite system contains three phases, namely the matrix of volume fraction V_m_ and tensile modulus E_m_, the dispersed filler of volume fraction $V_{f}^{\mathrm{dis}}$ and modulus $E_{f}^{\mathrm{dis}}$ and the agglomerated filler of volume fraction $V_{f}^{\mathrm{agg}}$ and modulus $E_{f}^{\mathrm{agg}}$. These three phases can be arranged in parallel or a series as shown schematically in Figure 2a,b. The strain is similar in all three in the case of a parallel arrangement of the three phases, while the stress is additive. In this case, the composite modulus is given by the modified parallel model (modified Voigt):

(11) $$E_{c}=V_{f}^{\mathrm{agg}}\cdot E_{f}^{\mathrm{agg}}+V_{f}^{\mathrm{dis}}\cdot E_{f}^{\mathrm{dis}}+\left(1-V_{f}^{\mathrm{agg}}-V_{f}^{\mathrm{dis}}\right)\cdot E_{m}$$

In the case of a series arrangement, the stress is similar in all three phases, as long as, the strain is additive, and the composite modulus can be determined from the modified series model (modified Reuss):

(12) $$E_{c}=\frac{E_{f}^{\mathrm{agg}}\cdot E_{f}^{\mathrm{dis}}\cdot E_{m}}{V_{f}^{\mathrm{agg}}\cdot E_{f}^{\mathrm{dis}}\cdot E_{m}+V_{f}^{\mathrm{dis}}\cdot E_{f}^{\mathrm{agg}}\cdot E_{m}+\left(1-V_{f}^{\mathrm{agg}}-V_{f}^{\mathrm{dis}}\right)\cdot E_{f}^{\mathrm{agg}}\cdot E_{f}^{\mathrm{dis}}}$$

where

(13) $$V_{f}^{\mathrm{agg}}=f\left(V_{f}\right)\cdot V_{f}$$

The volume fraction of the filler, $V_{f}^{\mathrm{agg}},$ is the percolating network. Chatterjee [46] proposed an equation for f(V_f_) above the percolation threshold of nanoparticles:

(14) $$f\left(V_{f}\right)=\{\begin{array}{c} 0,\;V_{f}<V_{c}\\ 1-e^{-A\cdot {\left(\frac{V_{f}}{V_{c}}-1\right)}^{0.474}},\;V_{f}\geq V_{c}\; \end{array}$$

where A is a constant parameter and V_c_ is the volume fraction of percolation threshold. f(V_f_) = 1 indicates that all nanoparticles are involved in the network and no filler is separately dispersed in the polymer matrix, while f(V_f_) = 0 indicates that the filler is dispersed in the polymer matrix. The volume fraction of nanoparticles dispersed within the matrix is proposed as:

(15) $$V_{f}^{\mathrm{dis}}=\left(1-f\left(V_{f}\right)\right)\cdot V_{f}$$

An equation can be suggested [40] for $E^{\mathrm{agg}}$ as:

(16) $$E^{\mathrm{agg}}=E_{f}\cdot {\left(V_{f}-V_{c}\right)}^{c}$$

where c is the percolation exponent. Accordingly, the best modulus is obtained by the lowest level of c which shows the inverse relation between the modulus level and the c factor as a percolation exponent. The c parameter depends on several parameters such as the density and strength/stiffness of the filler network in polymer nanocomposites. Paul [47] suggested a model for the composite containing dispersed particles as:

(17) $$E^{\mathrm{dis}}=E_{m}\cdot \frac{1+\left(h-1\right)\cdot V_{f}^{2/3}}{1+\left(h-1\right)\cdot \left(V_{f}^{2/3}-V_{f}\right)}$$

where h = Ef/Em.

Loos and Manas-Zloczower [48] assumed the network and dispersion of nanofillers above the percolation threshold and established the two forms of modified Takayanagi model. Figure 2c,d shows the series–parallel (model I) and parallel–series (model II) models. The modified Takayanagi model I is:

(18) $$E_{c}=\frac{\left(1-V_{f}\right)\cdot E_{m}\cdot E_{f}^{\mathrm{agg}}+\left(V_{f}-\Psi \right)\cdot E_{f}^{\mathrm{dis}}\cdot E_{f}^{\mathrm{agg}}}{\left(1-V_{f}\right)\cdot \Psi \cdot E_{m}+\Psi \cdot \left(V_{f}-\Psi \right)\cdot E_{f}^{\mathrm{dis}}+{\left(1-\Psi \right)}^{2}\cdot E_{f}^{\mathrm{agg}}}$$

where Ψ is the volume fraction of networked filler, and $E_{f}^{\mathrm{agg}}$ and $E_{f}^{\mathrm{dis}}$ are the Young moduli of regions containing networked and dispersed nanoparticles, respectively.

The modified Takayanagi model II is:

(19) $$E_{c}=\frac{\Psi \cdot \left(1-V_{f}\right)\cdot E_{f}^{\mathrm{dis}}\cdot E_{f}^{\mathrm{agg}}+\Psi \cdot \left(V_{f}-\Psi \right)\cdot E_{m}\cdot E_{f}^{\mathrm{agg}}+{\left(1-\Psi \right)}^{2}\cdot E_{m}\cdot E_{f}^{\mathrm{dis}}}{\left(1-V_{f}\right)\cdot E_{f}^{\mathrm{dis}}+\left(V_{f}-\Psi \right)\cdot E_{m}}$$

Ji et al. [50] suggested a three-phase micromechanical model following the Takayanagi homogenization approach taking into consideration the interfacial contribution. This model connects in parallel and series the matrix, the reinforcement, and the interphase. The high specific surface area of nanofillers leads to a large interfacial area between polymer matrix and nanofiller forming the third phase in polymer nanocomposites, known as interphase. Furthermore, the Takayanagi model’s series–parallel version provides efficient load transfer perpendicular to the applied stress and platelet reinforcement with a thickness t and both length and width ξ (with ξ » t). The Ji model is:

(20) $$\frac{E_{c}}{E_{m}}={\left[\left(1-\alpha \right)+\frac{\alpha -\beta }{\left(1-\alpha \right)+\alpha \cdot \frac{\left(h-1\right)}{\ln\left(h\right)}}+\frac{\beta }{\left(1-\alpha \right)+\frac{\left(\alpha -\beta \right)\cdot \left(h+1\right)}{2}+\frac{E_{f}}{E_{m}}\cdot \beta }\right]}^{-1}$$

With

(21) $$\{\begin{array}{c} \alpha =\sqrt{\left(2\cdot \frac{\tau }{t_{c}}+1\right)\cdot V_{f}}\\ \beta =\sqrt{V_{f}} \end{array}$$

where τ is the interphase thickness, h = E_i(0)/_E_m_ is the ratio of the interphase modulus on the surface of the particle, E_i(0)_, to that of the matrix E_m_. The most important parameters in this model are the thickness of particles t_c_, the thickness of the interface τ, and the stiffness ratio h which can affect the mechanical properties of the composite. When the influence of the interfacial region is negligible, the thickness of interface τ = 0, so that the parameter α = β = $\sqrt{V_{f}}$. In this case, the three-phase model is simplified to the conventional two-phase model of Takayanagi (series–parallel version).

### 3.2. Structural and Morphological Properties of HDPE/GNP Nanocomposites

**Figure 3:**
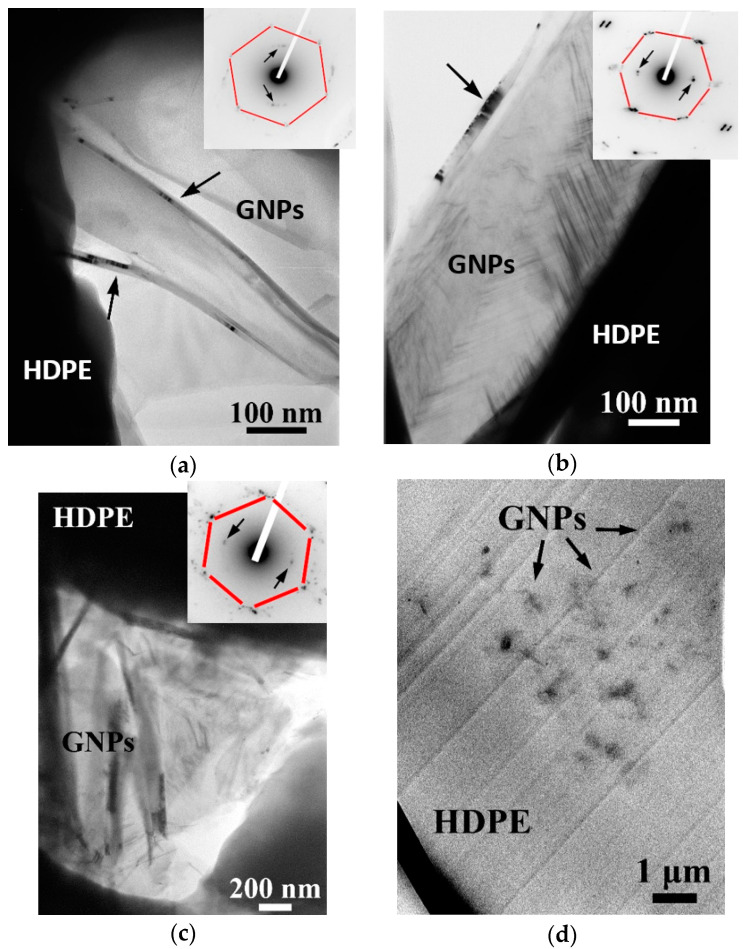
TEM micrographs of (**a**) High-Density Polyethylene (HDPE)/2.5 M5, (**b**) HDPE/2.5 M25, (**c**) HDPE/5 M5 nanocomposites showing a few planar overlapping Graphene nanoplatelets (GNPs), along with the (0001) zone axis of graphene. In the corresponding SAED patterns (inset), the hexagonal (0001) basal plane is evident, whereby the arrows denote reflections of buckled (0002) planes (**d**); low-magnification TEM micrograph of the HDPE/5 M25 nanocomposite, showing the formation of nanofiller aggregations within the matrix.

In Figure 3, TEM micrographs of thin HDPE/M5 and HDPE/M25 nanocomposite sections filled with 2.5 and 5 wt.% of GNPs are illustrated, showing planar graphene sheets effectively embedded within the HDPE matrix. Occasional buckling of the sheets may occur (marked with black arrows), giving rise to intense contrast of the (0002) planes of graphene. The wrinkled structure of graphene can improve the tensile strength because graphene acts as a crack propagation barrier and improves the mechanical interlocking between the polymer matrix and the filler [51]. The surface roughness and wrinkled nature of the GNPs cause interfacial interactions through the mechanical interlocking with polymer chains and hinder molecular mobility, increasing the temperature of glass transition (T_g_ values). This will also be confirmed in the following discussion. In the corresponding SAED patterns, the three families of {10-10} reflections belonging to the basal plane of 3–5 overlapping graphene sheets, slightly rotated about the (0001) axis, are observed, giving rise to the characteristic hexagonal shape of the basal plane, when viewed along with the (0001) projection direction (red lines guide the eye). Buckled (0002) crystal planes also contribute with the presence of 0002 reflections, denoted by arrows, in the SAED patterns of Figure 3.

As revealed from the micrographs of the HDPE nanocomposites at low filler concentration, HDPE/2.5 M5, and HDPE/2.5 M25, the GNPs are well dispersed in the matrix and they do not form large aggregates. However, HDPE/5 M5 and HDPE/5 M25 samples form irreversible agglomerates with increasing both the concentration and diameter size of GNPs. The melt-mixing process applied for the synthesis of HDPE/GNP nanocomposites cannot prevent the re-stacking and aggregation of GNPs because of the strong π–π interaction and van der Waals interaction between graphene sheets [52]. The high specific surface area of GNPs decreases and the effective load transfer between the HDPE matrix and the GNPs hinder, causing a significant decrease in the mechanical properties of the HDPE/GNPs nanocomposites, which is also confirmed in the following discussion.

TEM micrographs of nanocomposites filled with higher filler content show that fillers aggregate at high loadings with the GNP M25 buckling more easily, due to its larger diameter. In Figure 3d, a low-magnification TEM image of the HDPE/5 M25 composite is presented, revealing an inhomogeneous distribution of GNPs within the polymer matrix with a variety of differently sized nanofiller aggregations either embedded in those aggregates or interconnecting them. Additionally, some GNPs are isolated apart from the filler aggregates. From microscopy observations, it was found that while low filler concentrations allow a satisfactory dispersion, when the concentration and diameter size of GNPs increase, the HDPE nanocomposites are dominated by large filler aggregations. GNP agglomerates within the polymer matrix could cause structural imperfections such as holes and voids. The low elasticity of the HDPE/5 M25 sample, which will be discussed later, may be caused by the existence of these defects arising from the GNP M25 aggregation. In contrast, the efficient mechanical reinforcement with small-sized fillers at high filler content can be attributed to the combined effects of better dispersion, less agglomeration, and retention of the original filler morphology within the polymer matrix. Generally, the nanocomposites seem to present a rough surface providing a network-like structure in which the phonons can efficiently travel along and accelerate heat transfer. This leads to a significant increase in the thermal conductivity of the HDPE/GNP nanocomposites compared to neat HDPE [33].

**Figure 4:**
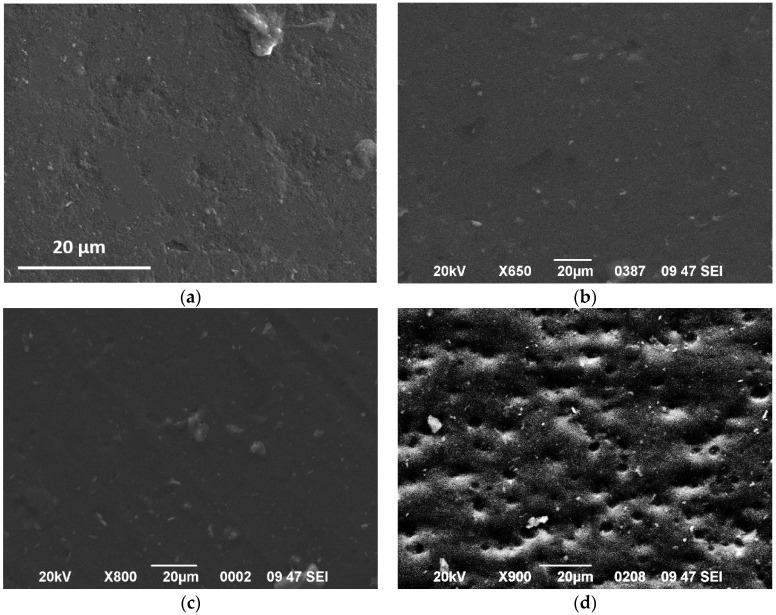
SEM images of surfaces of (**a**) HDPE/0.5 M5, (**b**) HDPE/0.5 M25, (**c**) HDPE/5 M5, and (**d**) HDPE/5 M25 nanocomposites.

Figure 4 shows SEM images of HDPE/0.5 M5, HDPE/0.5 M25, HDPE/5 M5, and HDPE/5 M25 nanocomposites. The surface of the HDPE/0.5 M5 was found to be almost smooth. The agglomerate size increases with increasing the filler content resulting in a competition between the reinforcing role of the GNPs and the GNPs’ content. The HDPE/5 M5 exhibits several GNPs protruding out of the surface of the polymer matrix. Two different dispersions of GNPs in HDPE are shown: separately dispersed and aggregated. From the SEM micrographs in Figure 4a–c, there are no observed voids between the HDPE matrix and the GNPs suggesting that the polymer melt bonded well with the GNPs. For this reason, the state of dispersion in the specimens indicates that a good interaction of the GNPs with the polymer matrix is achieved. When the diameter size increases, the surface of nanocomposites becomes rougher as microvoids can be seen due to GNP aggregates of the larger diameter size. These voids over the surface may prevent the entry of hardener into GNP aggregates and act as stress concentrations, leading to low adhesion and poor compatibility between GNP M25 and HDPE matrix and so, affecting the mechanical performance.

### 3.3. Mechanical and Thermomechanical Properties of HDPE/GNPs Nanocomposites

**Figure 5:**
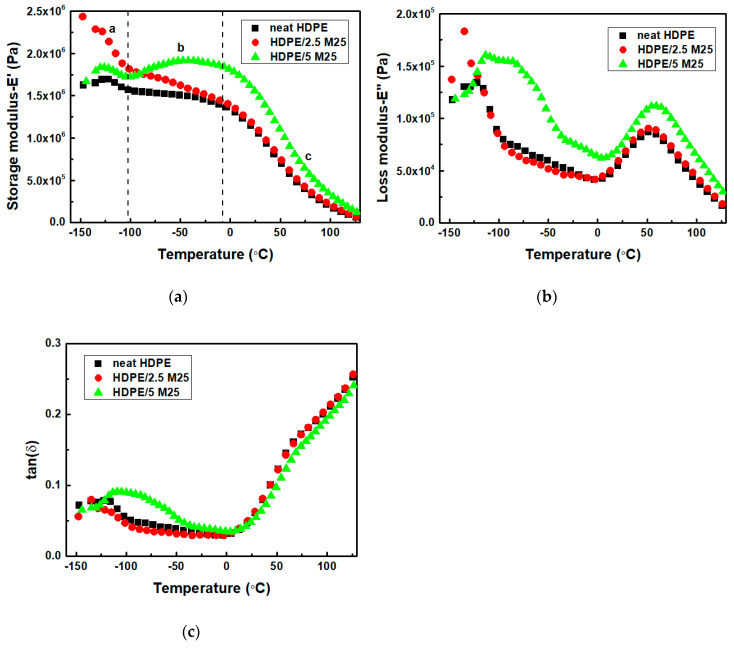
(**a**) Storage moduli, (**b**) loss moduli, and (**c**) tan(δ) values of HDPE/M25 nanocomposites filled 2.5, and 5 wt.% of GNPs.

**Figure 6:**
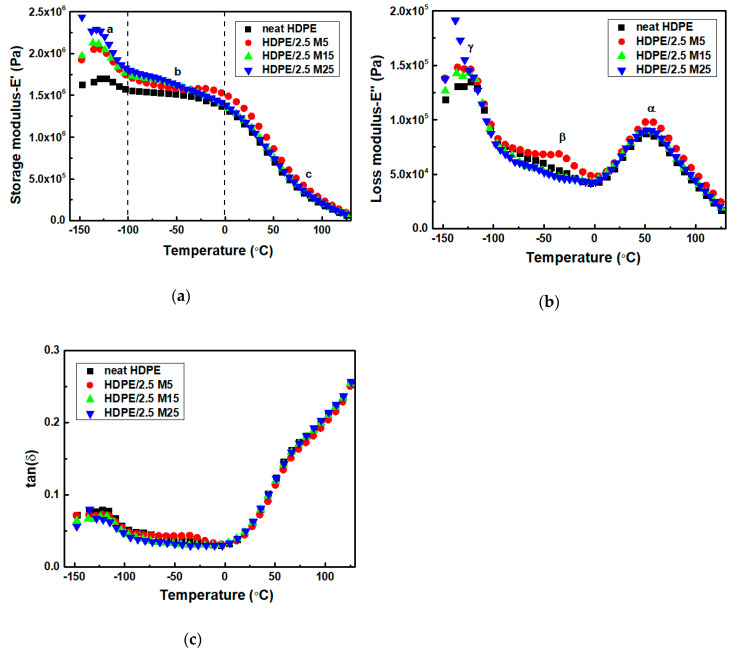
A comparative plot of (**a**) storage moduli, (**b**) loss moduli, and (**c**) tan(δ) values of HDPE/M5, HDPE/M15, and HDPE/M25 nanocomposites filled with 2.5 wt.% of GNPs.

Figure 5a, and Figures S4a and S5a of the supplementary information show the storage moduli values, E’, of HDPE/M25, HDPE/M5 and HDPE/M15 nanocomposites, respectively, as a function of temperature. The storage modulus values versus temperature plot is divided into three distinct regions of temperature for the HDPE/GNP nanocomposites as follows: (a) lower temperature range from −150 to −100 °C, (b) temperature range from −100 to 0 °C, and (c) temperature range from 0 to 130 °C. The HDPE/GNP nanocomposites show higher storage moduli values than neat HDPE over the whole temperature region indicating the mechanical reinforcing effect of GNPs, mainly in the low temperature region. In detail, the values of HDPE/2.5 M5, HDPE/2.5 M15, and HDPE/2.5 M25 nanocomposites are higher than those of neat HDPE and nanocomposites filled with 5 wt.% GNPs. On the one hand, the storage moduli of HDPE/M5 nanocomposites increase progressively in the low temperature region when the effective GNPs’ content increases from 2.5 to 5 wt.% and after that the E’ decreases much faster at higher temperatures. On the other hand, on increasing the GNPs’ content and size, the E´ values of HDPE/5 M15 and HDPE/5 M25 samples decrease much faster in the region (a) and after that, they increase. Therefore, the mechanical properties of the HDPE nanocomposites do not increase in proportion to the GNPs’ content. A decrease in E´ of PE composite content has been reported in the literature [53]. Figure 6a shows a comparative plot of HDPE/M5, HDPE/M15, and HDPE/M25 nanocomposites filled with 2.5 wt.% of GNPs. At low temperatures (−130 °C), the E´ of HDPE/2.5 M25 nanocomposite is higher compared to neat HDPE and the corresponding HDPE/GNP nanocomposites, confirming the reinforcement effect of GNP M25. The high storage modulus of GNPs increases the storage moduli values in the nanocomposites, restricting the mobility and resulting in a stiffened interphase. Additionally, the E’ values of neat HDPE and HDPE/GNP nanocomposites decrease significantly in the temperature range (c). Nevertheless, the E’ of HDPE/GNP nanocomposites are slightly higher than that of neat HDPE at all GNP compositions. The thermal movement of the HDPE matrix leads to a gradual reduction with increasing temperature. At 130 °C, the E’ values of neat HDPE and the HDPE/GNP nanocomposites were found to be almost equal because the HDPE matrix fully melts.

Loss modulus (E’’) reflects the amount of mechanical energy dissipated by the material corresponding to the viscous response. The curves of E’’ for neat HDPE and HDPE/GNP nanocomposites show three relaxation peaks at ~ −122, −40 and 55 °C, in Figure 5b and Figures S4b and S5b of the supplementary information. According to the literature [54], three relaxation processes can be found in PE samples: (i) the α-relaxation at high temperatures caused by the molecular motions in the crystalline regions; (ii) β-relaxation, which is attributed to the amorphous phase [55]; and (iii) γ-relaxation, the glass transition (Tg), the lowest temperature mechanical loss results from the local molecular motions in the amorphous part and the defects in the crystalline areas. The HDPE/M5 nanocomposites appear at three relaxations, α, β, and γ, while the HDPE/M15 and HDPE/M25 samples filled with lower filler content (0.5 and 2.5 wt.%) appear at two distinct transitions, namely, α and γ transitions. However, at higher filler content, the HPPE/5 M15 and HDPE/5 M25 nanocomposites appear at the α transition and a peak broadening effect of the β and γ transitions, confirming the mobility of the amorphous phase. According to Figure 6b, the E’’ value of the HDPE/2.5 M25 sample has a higher γ-relaxation compared to neat HDPE, restricting the mobility in the polymer chain. Additionally, the E’’ values of HDPE/2.5 M15 and HDPE/2.5 M25 nanocomposites decrease with an increase in the temperature range of β-relaxation compared to neat HDPE and HDPE/2.5 M5 samples because the molecular side chains in the interfacial amorphous region have sufficient energy to allow the movement of chains. The peak intensity and broadness of HDPE/M15 and HDPE/M25 nanocomposites increase with increasing the GNP concentration in the α-relaxation because of the decreased flexibility of the macromolecular chains in the surface layer and the poor interaction between the HDPE matrix and GNPs filler. So, the peaks of HDPE/2.5 GNP nanocomposites indicate that the polymer chains have higher energy dissipation ability whereas the broader peaks in the HDPE/5 M15 and HDPE/5 M25 nanocomposites indicate a distribution in terms of their relative chain mobility. It is evident that the incorporation of GNPs with a bigger diameter size has not only caused a restriction in mobility of the polymer chains but has also caused a relative distribution in the extent of the mobility of the polymer chains.

Figure 5c and Figures S4b and S5b of the supplementary information show the temperature dependence on the loss tangent and in fact, the glass transition temperature (Tg) can be calculated by using the peak temperature of tan(δ). It was found to be −122.4 °C, −122.4 °C, −118.5 °C, −117.4 °C, −115.2 °C, −110.6 °C, and −110.4 °C for neat HDPE, HDPE/2.5 M5, HDPE/2.5 M15, HDPE/2.5 M25, HDPE/5 M5, HDPE/5 M15, and HDPE/5 M25 nanocomposites, respectively. The increase in the γ relaxation temperature with increasing GNP size also suggests the presence of numerous GNP sheets, which affect polymer segmental motions, restricting the molecular mobility in the HDPE matrix. According to Figure 6c, the HDPE/2.5 GNP nanocomposites show lower tan(δ) intensity values than neat HDPE because of the increased stiffness of the nanocomposites, which is directly related to the damping ability; the viscoelastic energy dissipates less in the nanocomposites than in the neat polymer. For HDPE/5 M15 and HDPE/5 M25 nanocomposites, the tan(δ) is considerably higher than that of the neat HDPE in the low temperature range, revealing similar results with the storage and loss moduli study. Therefore, the mobilization of the polymer chains requires more energy, resulting in higher T_g_ values. As for the α-relaxation, it has been linked to PE crystalline mobility. The α-relaxation temperature of neat HDPE, HDPE/2.5 M5, HDPE/2.5 M15, HDPE/2.5 M25, HDPE/5 M5, HDPE/5 M15, and HDPE/5 M25 nanocomposites was found to be 50.6 °C, 52.1 °C, 53.1 °C, 57.1 °C, 53 °C, 55.3 °C, and 61.2 °C, respectively. Higher α-transition temperature for the HDPE/GNPs nanocomposites suggests a rigid crystal phase with less mobile interfaces [55].

The higher values of storage and loss moduli for HDPE/2.5 M25 nanocomposite leads to a reduction in the intensity values of tan(δ) in Figure 6c because of the formation of the interfacial bonding between the HDPE matrix and the GNPs M25 filler. The stiffness improves because of the higher stiffness values of the GNPs as opposed to increased crystallinity in the HDPE/GNP nanocomposites [56]. This agrees with the reported results in the literature [57], in which the incorporation of rigid fillers into a polymer matrix limits the molecular mobility and increases the material viscosity. The behavior is also consistent with the tensile tests, which will be discussed later.

**Figure 7:**
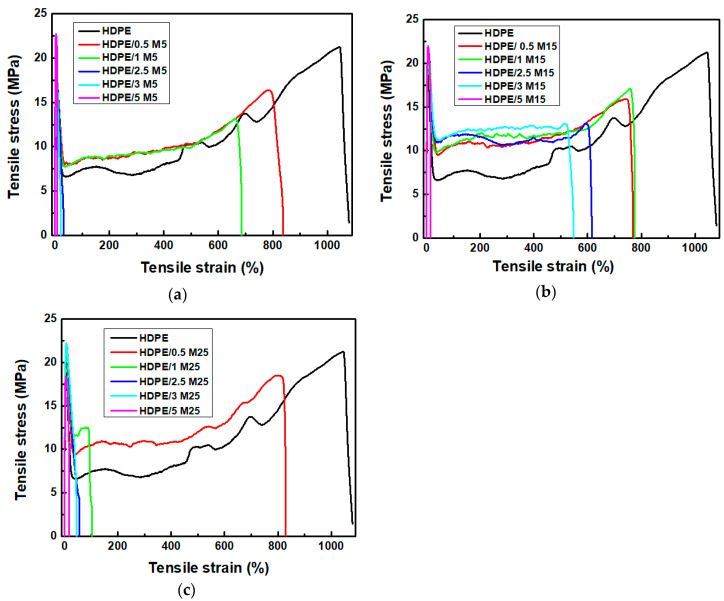
Typical stress–strain curves of (**a**) HDPE/M5, (**b**) HDPE/M15, and (**c**) HDPE/M25 nanocomposites at various GNP contents.

The mechanical properties of the HDPE nanocomposites filled with GNPs of various diameter sizes were studied by tensile tests. Figure 7 shows the stress–strain curves of the neat HDPE, HDPE/M5, HDPE/M15, and HDPE/M25 nanocomposites. The values of tensile stress at yield and Young moduli of HDPE/M5, HDPE/M15, and HDPE/M25 nanocomposites were found to be higher than those of neat HDPE. GNP M25 with the larger diameter size cause a stronger increase in the tensile stress at yield values than those of smaller diameter size (GNP M5 and GNP M15) until a concentration of 3 wt.%. After that, the HDPE/M5 sample filled with 5 wt.% of GNPs presents higher values for tensile stress at yield and Young modulus, 22.6 ± 0.5 MPa and 867.7 ± 19.1 MPa, respectively, than HDPE/5 M15 and HDPE/5 M25 nanocomposites. Chen et al. [57] also reported a reduction in the tensile stress at yield values of HDPE-expanded graphite nanocomposites suggesting a decrease in the mobility of the HDPE molecules. Additionally, GNPs cause a significant decrease in the elongation at break for HDPE/GNP nanocomposites. It should be noticed that the response of the HDPE/GNP nanocomposites changes from ductile to brittle behavior with increasing GNPs content.

**Figure 8:**
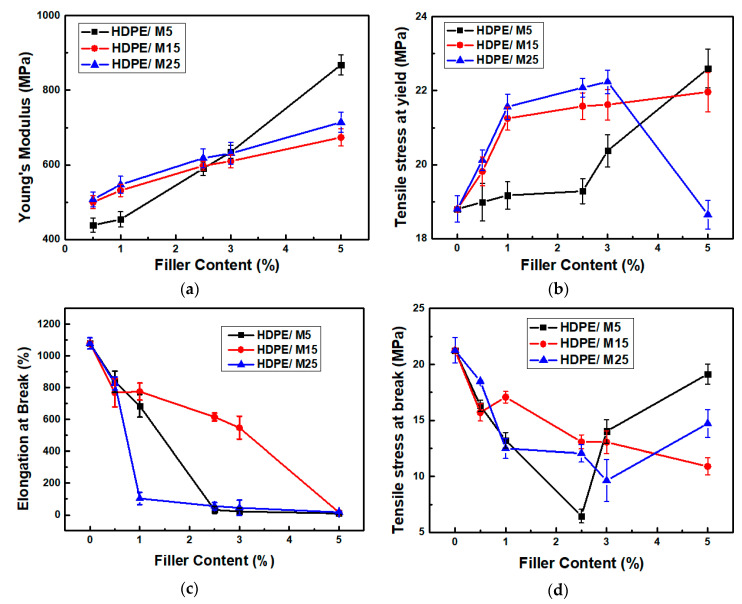
(**a**)Young modulus, (**b**) tensile stress at yield, (**c**) elongation at break, and (**d**) tensile stress at break of GNPs M5, M15, and GNP M25-filled HDPE nanocomposites as a function of GNP content.

Figure 8 shows the extracted values of the Young modulus, tensile stress at yield, tensile stress at break, and elongation at break for HDPE/M5, HDPE/M15, and HDPE/M25 nanocomposites as a function of GNP content. The Young moduli and tensile stress at the yield of HDPE/GNP nanocomposites increase significantly with increasing GNPs content. The Young moduli increase by about 5%, 23%, and 27% for HDPE/M5, HDPE/M15, and HDPE/M25 nanocomposites filled with 1 wt.% of GNPs, respectively, compared to the neat HDPE. Similarly, the Young moduli of HDPE/M5, HDPE/M15, and HDPE/M25 nanocomposites show a substantial increase by about 100%, 55%, and 65%, respectively, with increasing the GNP content at 5 wt.%. The high intrinsic mechanical characteristics and the large aspect ratio of the 2-D surface of GNPs lead to good interfacial stress transfer. Therefore, there is an improvement in the mechanical properties of the HDPE/GNP nanocomposites. The values of the Young moduli and tensile stress at yield of HDPE/M25 nanocomposites are higher than those of neat HDPE, HDPE/M5, and HDPE/M15 nanocomposites for the same GNP content until 3 wt.%. So, the large aspect ratio and platelet size of GNP M25 are beneficial for stress transfer further increasing the values of the Young moduli and tensile stress at yield at low concentrations compared to nanocomposites with a lower diameter size [58]. However, at higher filler loading (5 wt.% GNPs), the increase in the effective reinforcement of Young’s modulus and tensile stress at yield was found to be lower than HDPE/M5 nanocomposite, nonetheless, the Young modulus of HDPE/M25 nanocomposite is still higher than HDPE/M15 sample. The stress transfer in the HDPE/GNP nanocomposites has been suppressed at higher GNP content due to GNP agglomerates. As shown in Figure 3, GNPs restack together in layers due to van der Waals forces and π–π attraction between GNP planes [59]. A more uniform interfacial stress transfer was found for the aggregated GNPs in the HDPE/5 M5 nanocomposite minimizing the stress concentration centers. In this case, the Young modulus and tensile stress at yield values of HDPE/5 M5 nanocomposites increase.

Figure 8c,d show a decrease in the values of elongation at break and tensile stress at break with increasing filler content. The HDPE nanocomposites become significantly stiffer. Indeed, Figure 8c shows that the neat HDPE presents a wide extension by activating necking and cold-drawing mechanisms, while HDPE/GNP nanocomposites have a very small plastic area, resulting in a brittle behavior immediately after the yielding point. In detail, the elongation at break decreases from 1079% for neat HDPE to 7%, 12%, and 10% for HDPE/5 M5, HDPE/5 M15, and HDPE/5 M25, respectively. King et al. [60] have reported a strong decrease in the elongation at break for PC/GNP M5 material. Figure 8d shows a decrease in the stress at the break for all HDPE/GNP nanocomposites due to low interfacial adhesion between the polymer and filler; the material loses its toughness. In detail, the values of tensile stress at break decrease from 21 ± 1.1 MPa for the neat HDPE, to about 19 ± 0.9 MPa, 11 ± 0.8 MPa, and 15 ± 1.3 MPa for HDPE/5 M5, HDPE/5 M15, and HDPE/5 M25 nanocomposites, respectively.

The tensile stress at yield of the HDPE nanocomposites requires strong filler/matrix bonding, while the micro-deformation mechanism in the elongation at the break depends on the presence of GNP agglomerates in the HDPE matrix. The HDPE nanocomposites become stiffer with increasing GNP content and size. This confirms the increase in the elastic modulus, the decrease in the elongation at break, and the shift of the Tg peak to higher values. So, the tensile properties of HDPE/GNP nanocomposites were found to be influenced by the dispersibility of GNPs in the HDPE matrix and the interfacial interaction between GNPs and the HDPE matrix.

### 3.4. Micromechanical Modeling of HDPE/GNP Nanocomposites

**Figure 9:**
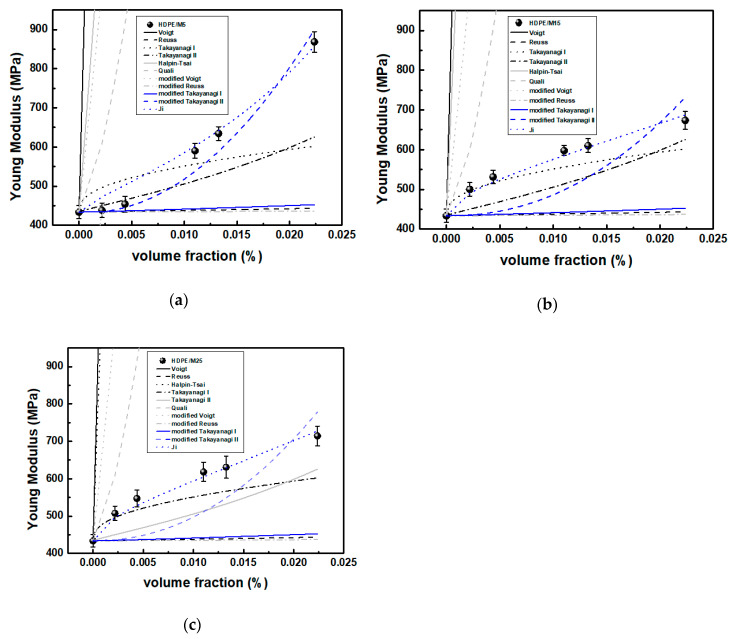
Theoretical modeling of elastic modulus for (**a**) HDPE/M5, (**b**) HDPE/M15, and (**c**) HDPE/M25 nanocomposites as a function of GNP content using various models based on micromechanics and composite theories.

Μicromechanical-based models have been used to study the effect of filler size on the mechanical properties of the HDPE matrix and compare them to the experimental data through tensile tests. Figure 9. shows both the experimental results and theoretical predictions for the HDPE/GNP nanocomposites. In all cases, the theoretical predictions indicate that the incorporation of GNPs into the HDPE matrix leads to an enhancement in the mechanical properties of nanocomposites. The conventional models such as the Voigt model, Reuss model, and Halpin–Tsai model, generally used in the literature [27], cannot predict the HDPE/GNP nanocomposites well. The high stiffness of the GNPs compared to the HDPE matrix, make it difficult for accurate predictions through the Halpin–Tsai model [42] which takes into account the aspect ratio and volume fraction of the fillers. In this work, the experimental results were found to be dependent on mobility in the interfacial region of HDPE nanocomposites. This suggests that the experimental results are influenced by other parameters.

Ouali et al. [44] proposed a power-law model above the mechanical percolation threshold for the moduli of polymer composites. In this work, the percolation concept in the series–parallel and parallel–series models of Takayanagi [37] was used to predict the mechanical properties of the HDPE/GNP nanocomposites. As mentioned earlier, GNPs tend to aggregate and form a 3-D network within the polymer matrix above a critical concentration [61]. According to the results presented in Table 1, the percolation threshold, Vc, is inversely correlated to the aspect ratio of GNPs. On the one hand, the higher V_c_ value of GNP M5 in HDPE/M5 nanocomposites demonstrates that a greater number of GNPs are necessary to reach the percolation threshold. On the other hand, the lower V_c_ value of GNP M25 in the HDPE/M25 nanocomposites form a filler network at a smaller concentration of GNPs. In this way, the diameter size of GNPs affects the concentration of networked GNPs in the HDPE nanocomposites significantly. Above the percolation threshold, the matrix should mainly involve the filler into the 3-D network, although the matrix may still contain some well dispersed or agglomerated GNPs. Therefore, a simple mechanical model can be formed including the matrix, the well dispersed GNPs, and the agglomerated GNPs [48]. All these constituents have different mechanical properties and they will contribute to the overall mechanical behavior of the composite system.

Figure 9a shows that the modified Takayanagi model II fit the experimental values of the moduli of the HDPE/M5 nanocomposites at low filler content well. Table 1 lists the fitting parameters such as parameter A of the switching function and percolation exponent c. The mechanical behavior of HDPE/M5 nanocomposites at higher filler contents was found to be dependent on the mobility of macromolecules in the interfacial region because the E_agg_ modulus increases at higher GNP content. The stronger network, formed at 5 wt.% filler content, causes a stiff nanocomposite, promoting the modulus of nanocomposite to higher levels. So, the Ji model is the micromechanical model that predicts the effective reinforcement of the HDPE/M5 nanocomposites at higher filler contents well. As mentioned earlier, the Young modulus of HDPE/M5 nanocomposite filled with 5 wt.% of GNPs has higher values compared to HDPE/5 M15 and HDPE/5 M25 nanocomposites. The change in mechanism from Takayanagi II to the Ji model also confirms the higher value of the Young modulus for the HDPE/5 M5 sample.

On the one hand, the inclusion of the percolation concept in the series–parallel and parallel–series models of Takayanagi can predict the mechanical behavior of HDPE systems filled with the small diameter size and content. On the other hand, Figure 9b and Figure 8c show that the Ji model predicts the Young moduli of HDPE/M15 and HDPE/M25 nanocomposites with the larger diameter size well. Table 1 also lists the thickness of the interfacial region (τ) and the modulus of the interfacial region (Ei(0)). The large diameter size of GNP M25 causes a good interfacial interaction between polymer chains and GNPs increasing the modulus at low filler content. Both the network density and the amount of dispersed GNP M25 into the HDPE matrix increase with further increasing the GNP content, confirming the formation of thick and strong interphase in HDPE/GNP nanocomposites. The Ei values also confirm that the well-dispersed and agglomerated GNPs form a thick interphase around them. The HDPE/M25 sample presents thicker interphase than that of the HDPE/M15 nanocomposite. At lower GNP M25 content, the modulus of the GNP controls the reinforcement mechanism in HDPE nanocomposites, while the amount of nanofiller, and so the distance between adjacent GNPs, influences the mechanical response by increasing the GNP content further and above V_c_. However, when the filler loading is high enough, the reinforcing efficiency decreases because the HDPE matrix cannot control the number of GNP agglomerates formed into the matrix. 

### 3.5. Fractography of HDPE/GNPs Nanocomposites

**Figure 10:**
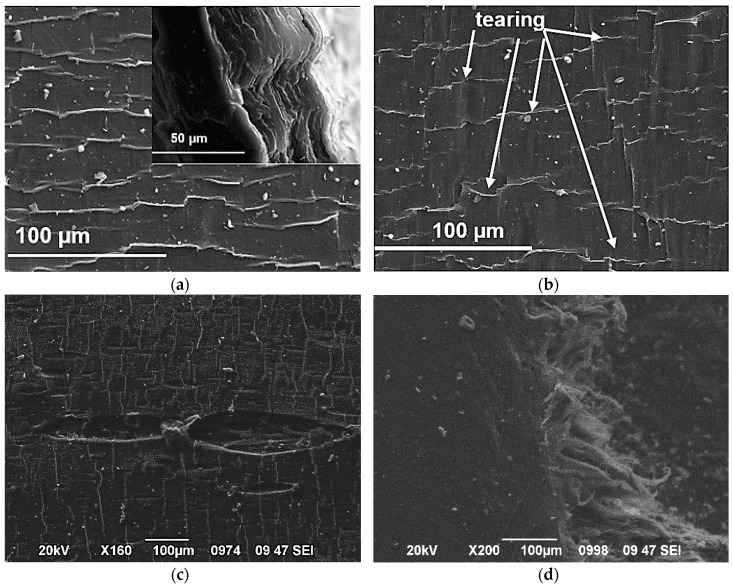

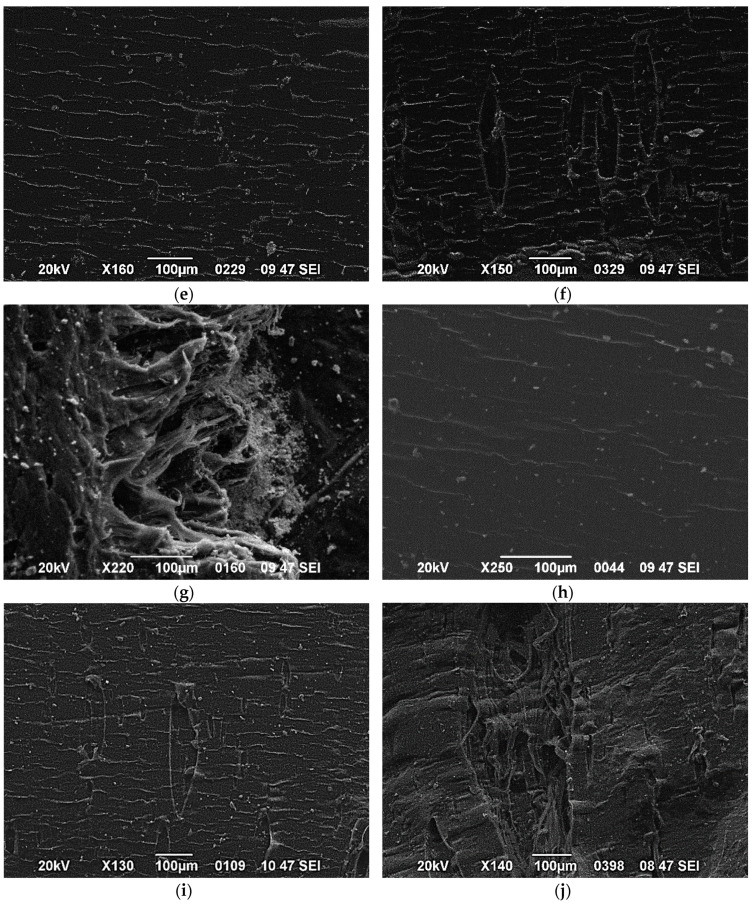
Fractured specimens of (**a**) neat HDPE, (**b**) HDPE/0.5 M5, (**c**) HDPE/2.5 M5, (**d**) HDPE/5 M5, (**e**) HDPE/0.5 M15, (**f**) HDPE/2.5 M15, (**g**) HDPE/5 M15, (**h**) HDPE/0.5 M25, (**i**) HDPE/2.5 M25, and (**j**) HDPE/5 M25 nanocomposites.

Fractography is an indispensable tool for understanding the fracture behavior and toughening mechanisms of composites. The tensile testing findings indicate that the HDPE/GNP nanocomposites become more brittle with increasing both the GNP content and size. The deformation of neat HDPE and HDPE/GNP nanocomposites includes deformation bands, crazing, tearing, microcracking, and regular cracking [62]. Figure 10 shows the failure surface of neat HDPE and HDPE/GNP nanocomposites after tensile tests. Figure 10a displays the typical shear band morphology and fracture features of neat HDPE [63]. Figure 10a (inset) shows striations formed on the fracture surface of neat HDPE. The addition of 0.5 wt.% and 2.5 wt.% GNPs in the matrix is not enough to change the surface morphology of neat HDPE as shear banding is the predominant mode of tensile deformation for nanocomposites. As a result, a ductile fracture occurs in the neat HDPE, HDPE/0.5 GNPs, and HDPE/2.5 GNP nanocomposites. However, the presence of voids and the initiation of cracks from these voids can be noticed from the SEM micrographs of nanocomposites samples filled with 2.5 wt.% of GNPs. In this case, the voids become stabilized by fibrils of polymeric material. At lower loads and under identical test conditions, the degree of tearing was found to be less pronounced for HDE/GNPs M25 nanocomposites. The significantly reduced surface damage behavior can be attributed to the reinforcement of HDPE filled with GNP M25 at low concentrations.

The fracture mechanism for HDPE nanocomposites filled with GNPs at high filler content, 5 wt.%, is different from the HDPE/GNP nanocomposites at small filler content. Here the tearing process involves fibrillation, presumably through extensive localized plastic deformation. Crazes grow and increase in number with increasing diameter sizes. Figure 10j shows the fracture surface of the HDPE/5 M25 nanocomposite with a 600 µm crack opening along with other smaller cracks and the unexpected cutting of the fibrils of the material. So, the surfaces of HDPE nanocomposites filled with GNP M25 were found to be rough with large and deep voids at the concentration of 5 wt.%. The high filler aggregates found in HDPE/5 M25 nanocomposite serve as stress concentrators leading to an early failure. The fracture surface of HDPE/5 M25 nanocomposite becomes brittle and the load applied to be transferred through the interactions will become less efficient reducing tensile strength and causing a ductile to a brittle transition. The mechanism that controls the ductile fracture of HDPE/GNP nanocomposites at low filler content is the shear banding, while the mechanism for more brittle nanocomposites with higher diameter size and GNPs content is the crazing fracture.

**Figure 11:**
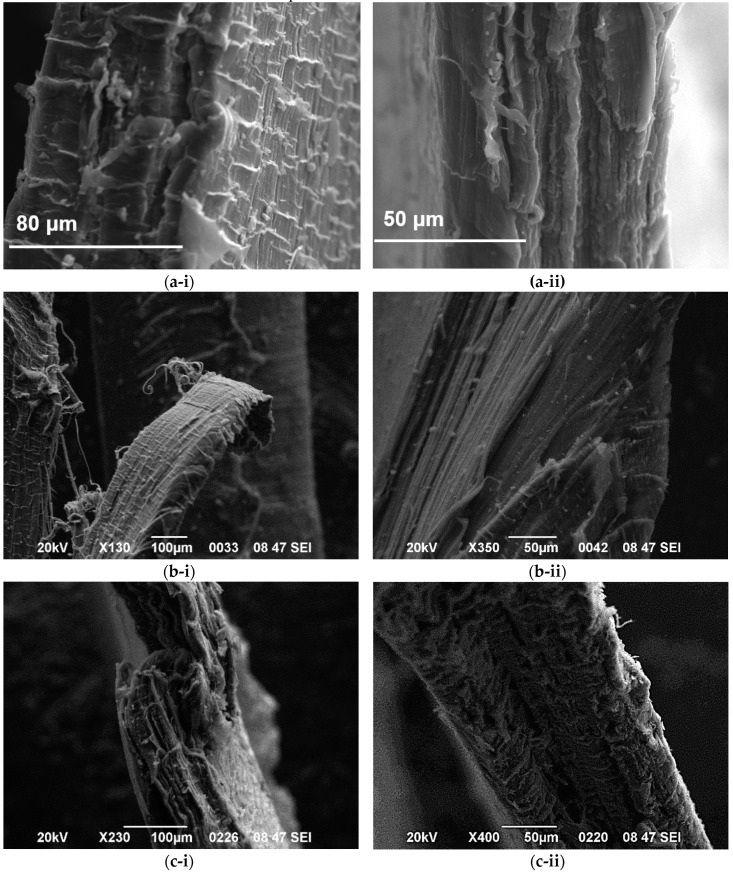
Fibril deformation for (**a**) HDPE/0.5 M5, (**b**) HDPE/0.5 M15, and (**c**) HDPE/0.5 M25 nanocomposites with increasing magnification from left to right, 100 μm and 50 μm.

**Figure 12:**
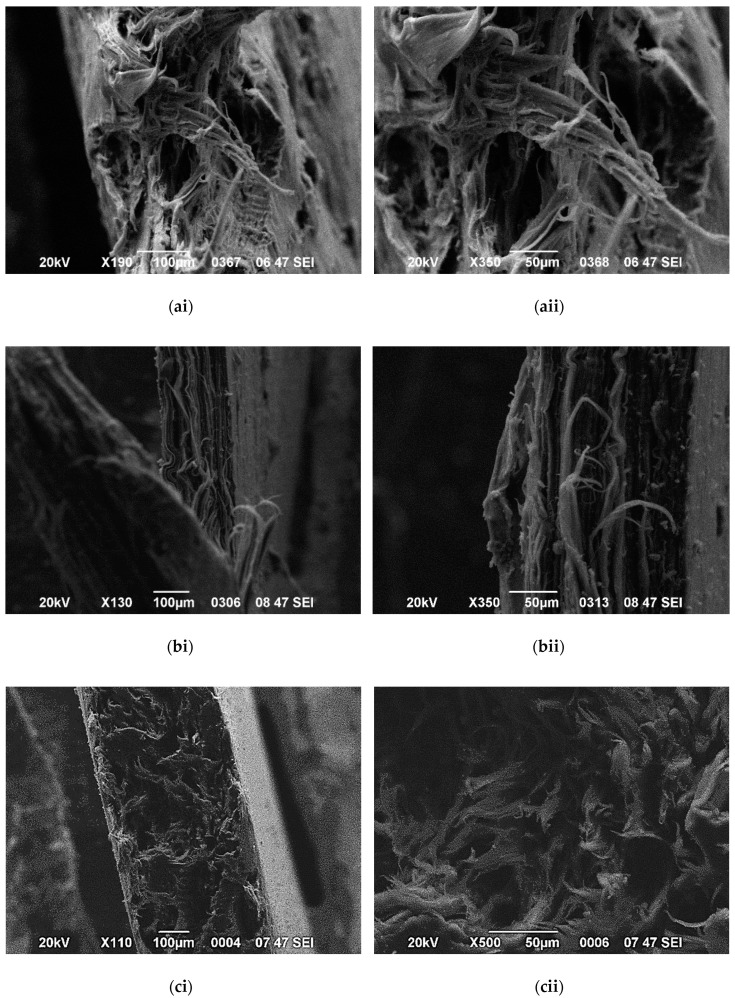
Fibril deformation for (**a**) HDPE/5 M5, (**b**) HDPE/5 M15, and (**c**) HDPE/5 M25 nanocomposites with increasing magnification from left to right, 100 μm and 50 μm.

The change in fibrillar density depends on the filler content and diameter size. For the low GNP content, the addition of 0.5 wt.% GNPs in the HDPE matrix does not change the morphology of the matrix as highly elongated fibrils are shown in Figure 11. However, the fibrils were unexpectedly cut with a lower degree of deformation with increasing GNP content, suggesting a premature failure and a brittle fracture performance. In this case, crazing becomes the principle deformation mechanism as already mentioned above [64]. Figure 12 shows a difference in the nanocomposites filled with 5 wt.% because shorter fibers can be seen in the fracture surface of the HDPE/5 M25 nanocomposite compared to HDPE/5 M5 and HDPE/5 M15 samples. This indicates that the large aggregates found in the HDPE/5 M25 nanocomposite inhibit the formation of the HDPE fiber morphology because GNP M25 act as stress concentrators, promoting the crazing process. Therefore, the fracture surface of HDPE nanocomposites filled with higher filler size and content shows much less plastic deformation than HDPE/GNP nanocomposites with lower GNP size and content.

## 4. Conclusions

This study provides an insight into the mechanical behavior of HDPE nanocomposites filled with various diameter sizes and in this way, important information for the processing and the design of thermally conductive polymeric materials with well-controlled properties. It was found that the presence of GNPs improves the tensile modulus and tensile strength of the HDPE/GNP nanocomposites and decreases the elongation at break and tensile stress at break. A homogeneous dispersion can be found only in HDPE nanocomposites at low filler content, while large aggregates are formed with increasing the GNP content, leading to more brittle failure. Both the morphological and microstructural characterizations demonstrate that the presence of GNP M25 aggregates creates a conductive path in the HDPE nanocomposites, resulting in better interfacial stress transfer between GNPs and HDPE at low filler content. The conventional models of polymer composites for the prediction of the tensile modulus do not accurately predict the behavior of HDPE/GNP nanocomposites because of the morphological complexities presented in these nanomaterials. In general, over-estimation or under-estimation of the material property is observed due to simplistic model assumptions. The three-phase model proposed by Ji agrees with experimental results for HDPE nanocomposites filled with high GNP content and size, while the Takayanagi modified model II correlates with the experimental results of HDPE/M5 nanocomposites at low filler content. Macroscopically, shear banding is the predominant deformation mechanism at low filler contents leading to the higher ductility of the samples, while crazing is the fracture mechanism for brittle HDPE/GNP nanocomposites at higher GNP content.

## Figures and Tables

**Table 1:** Calculated mechanical parameters of HDPE/GNP nanocomposites.

Mechanical Model	A/A	Sample		
		HDPE/M5	HDPE/M15	HDPE/M25
	V_c_ (vol.%)	0.0018	0.0006	0.0004
**Modified Takayanagi II Model**	c	0.60	-	-
**Ji Model**	τ (nm)	1686.9	20.3	25.8
	E_i(0)_ (GPa)	0.47	6.42	1.37

## Supplementary Materials

The following are available online at https://www.mdpi.com/2073-4360/12/8/1719/s1, Figure S1: Schematic diagram of melt mix processing of HDPE/GNPs nanocomposites, Figure S2: The principle of DMA measurements for HDPE/GNPs nanocomposites, Figure S3: The tensile tests experiment setup for HDPE/GNPs nanocomposites, Figure S4: Storage moduli, (**b**) loss moduli, and (**c**) tan(δ) values of HDPE/M5 nanocomposites, Figure S5: Storage moduli, (**b**) loss moduli, and (**c**) tan(δ) values of HDPE/M15 nanocomposites.
