# Supplementary material for: Influence of Graphene Platelet Aspect Ratio on the Mechanical Properties of HDPE Nanocomposites: Microscopic Observation and Micromechanical Modeling
Source: Polymers (Basel). 2020 Jul 31;12(8):1719. doi: 10.3390/polym12081719 (PMC7463753; doi:10.3390/polym12081719)
Supplement: Supplementary file 1 [file polymers-12-01719-s001.pdf]

polymers

Supplementary File

Influence of graphene platelet aspect ratio on the mechanical properties of HDPE nanocomposites: Microscopic observation and micromechanical modeling

Evangelia Tarani, Iouliana Chrysafi, Alfréd Kállay-Menyhárd, Eleni Pavlidou, Thomas Kehagias, Dimitrios N. Bikiaris, George Vourlias, and Konstantinos Chrissafis

Supplementary Information

S1. Experimental Methods

HDPE nanocomposites containing different sizes (5 μm, 15 μm, and 25 μm in diameter) and various loading levels ranging from 0.1 to 5 wt. % (0.5, 1, 2.5, 3 and, 5 wt. % of GNPs) were prepared by melt mixing. Figure S1 shows a schematic diagram of the melt mixing process of HDPE/GNPs nanocomposites.

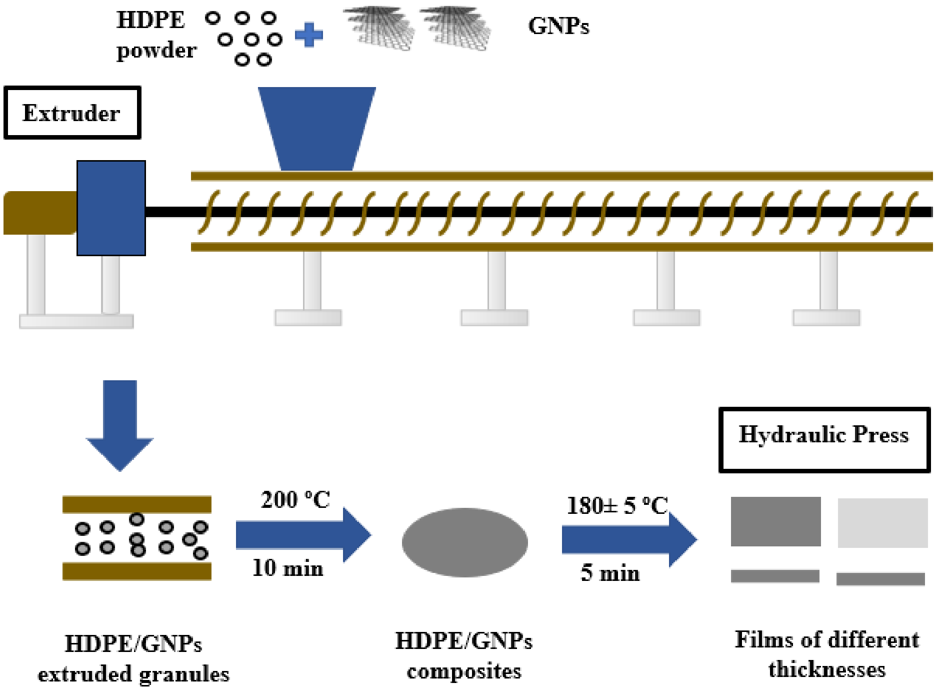

Figure S1. Schematic diagram of melt mix processing of HDPE/GNPs nanocomposites.

Dynamic mechanical analysis (DMA) is a useful tool for measuring the material properties in the solid state under dynamic conditions as a function of temperature and understanding the mechanical behavior of the polymeric systems at molecular level. Figure S2 shows the principle of DMA experiments used for the study of HDPE/GNPs nanocomposites. A complex sinusoidal stress is applied to the sample in the DMA experiments. The stress strain relationship is:

$$\sigma = \varepsilon_0 E' \sin \omega t + \varepsilon_0 E'' \cos \omega t \quad (1)$$

$$E' = \left(\frac{stress}{strain}\right) \cdot \cos \delta \quad (2)$$

$$E'' = \left(\frac{stress}{strain}\right) \cdot \sin \delta \quad (3)$$

$$\tan(\delta) = \frac{E''}{E'} \quad (4)$$

where ω = period of strain oscillation or angular frequency, t = time and δ = phase difference between stress and strain, loss angle.

The dynamic response of the sample results in the elastic and viscous components. The dynamic storage modulus (E') describes the in-phase component and it indicates the inherent stiffness of the material. The loss modulus (E'') describes the out of phase component and it gives the amount of energy dissipated as heat during the deformation.

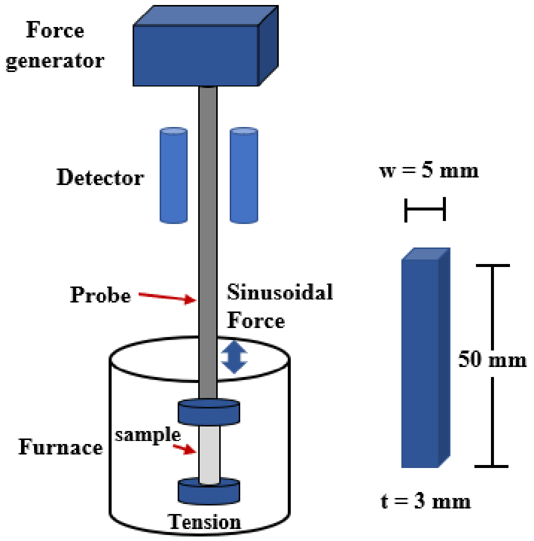

Figure S2. The principle of DMA measurements for HDPE/GNPs nanocomposites.

During tensile testing, a "dog-bone" sample is placed in the grips of movable and stationary fixtures in an Instron device. During the procedure, the samples are pulled until the fracture and so the applied load versus the elongation of the sample is measured. Figure S3 shows the tensile tests experiment setup for HDPE/GNPs nanocomposites.

During stretching the sample, the amount of force (F) applied is measured, and then, stress (σ) is obtained by dividing the force by the cross-section area (A) of the sample.

$$\sigma = \frac{F}{A} \quad (5)$$

Strain (E) is defined according to the equation below:

$$\varepsilon = \frac{\Delta L}{L_0} \quad (6)$$

where ΔL is the change in gage length of specimen and L_0 the initial gage length. Percent elongation (%E) is the extension at the break by the original gage length, multiplied by 100.

$$\%\ elongation = \frac{\Delta L}{L_0} \times 100 \quad (7)$$

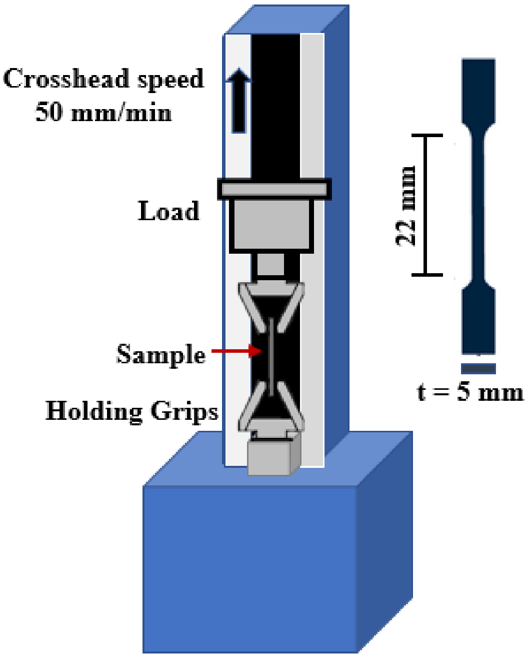

Figure S3. The tensile tests experiment setup for HDPE/GNPs nanocomposites.

S2. Thermomechanical Properties of HDPE/GNPs nanocomposites

Dynamic Mechanical Analysis (DMA) measurements were then carried out to provide supporting information about the dispersion and network formation of GNPs in the HDPE/GNPs nanocomposites. Figure S4 and Figure S5 show the storage moduli (E′), the loss moduli (E′′) and tan(δ) values of neat HDPE, HDPE/M5 and HDPE/M15 nanocomposites filled with 2.5 wt.% and 5wt.% of GNPs as a function of temperature.

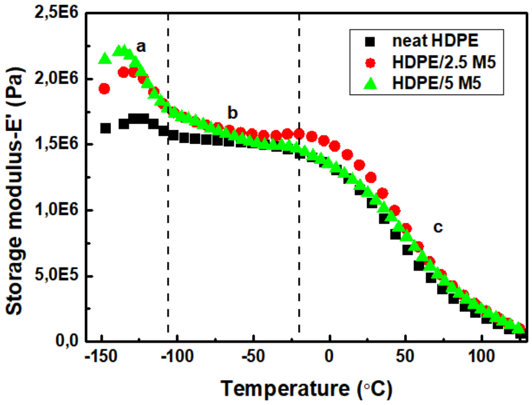

(**a**)

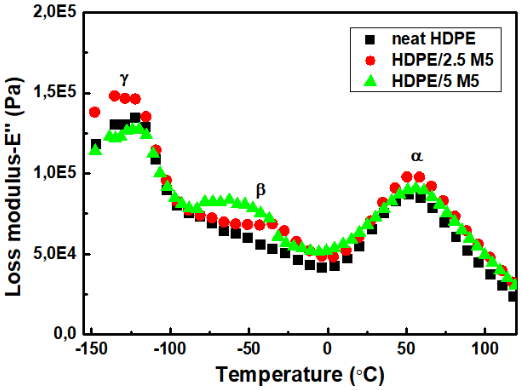

(**b**)

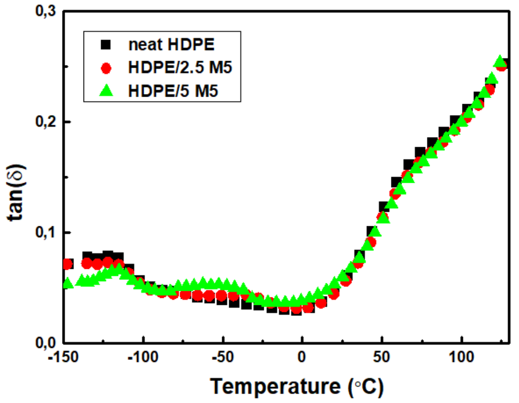

(c)

Figure S4. (**a**) Storage moduli, (**b**) loss moduli, and (**c**) tan(δ) values of HDPE/M5 nanocomposites.

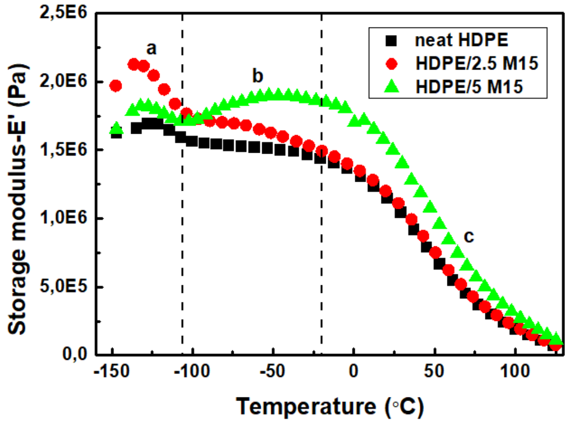

(a)

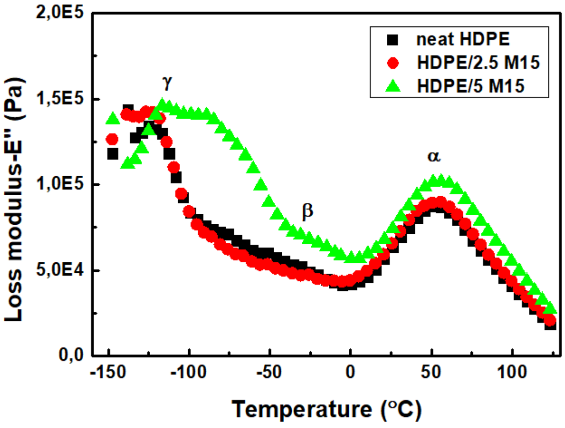

(b)

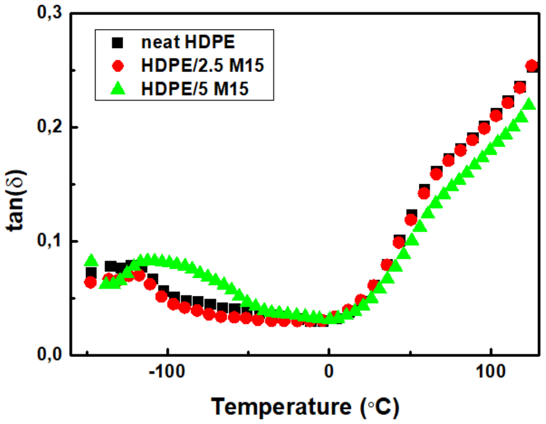

(c)

Figure S5. (**a**) Storage moduli, (**b**) loss moduli, and (**c**) tan(δ) values of HDPE/M15 nanocomposites.
